# An exploration of potential output measures to assess efficiency and productivity for labour and birth in Australia

**DOI:** 10.1186/s12884-021-04181-x

**Published:** 2021-10-19

**Authors:** Bonnie Eklom, Sally Tracy, Emily Callander

**Affiliations:** 1 College of Public Health, Medical and Veterinary Science, James Cook University, 1 James Cook Drive, Townsville, Queensland 4811 Australia; 2 The School of Public Health and Preventative Medicine, Monash University, Melbourne, Australia; 3 The Molly Wardaguga Research Centre, Charles Darwin University, Darwin, NT Australia; 4 The University of Sydney, 88 Mallett Street, Camperdown, NSW 2050 Australia

**Keywords:** Maternity, Efficiency and productivity, Maternity outcome measures

## Abstract

**Background:**

In maternity services, as in other areas of healthcare, increasing emphasis is placed on improving “efficiency” or “productivity”. The first step in any efficiency and productivity analysis is the selection of relevant input and output measures. Within healthcare quantifying what is produced (outputs) can be difficult.

The aim of this paper is to identify a potential output measure, that can be used in an assessment of the efficiency and productivity of labour and birth in-hospital care in Australia and to assess the extent to which it reflects the principles of woman-centred care.

**Methods:**

This paper will survey available perinatal and maternal datasets in Australia to identify potential output measures; map identified output variables against the principles of woman-centred care outlined in Australia’s national maternity strategy; and based on this, create a preliminary composite outcome measure for use in assessing the efficiency and productivity of Australian maternity services.

**Results:**

There are significant gaps in Australia’s maternity data collections with regard to measuring how well a maternity service is performing against the values of respect, choice and access; however safety is well measured. Our proposed composite measure identified that of the 63,215 births in Queensland in 2014, 67% met the criteria of quality outlined in our composite measure.

**Conclusions:**

Adoption in Australia of the collection of woman-reported maternity outcomes would substantially strengthen Australia’s national maternity data collections and provide a more holistic view of pregnancy and childbirth in Australia beyond traditional measure of maternal and neonate morbidity and mortality. Such measures to capture respect, choice and access could complement existing safety measures to inform the assessment of productivity and efficiency in maternity care.

## Background

In maternity services, as in other areas of healthcare, increasing emphasis is placed on improving “efficiency” or “productivity” [1, 2]. These terms are sometimes seen as synonymous with cost-cutting, and those on the frontline of delivering care may feel that the terms are used as a means of facilitating the reduction of resources with little concern for how that effects the quality of care [3, 4]. Such scenarios are actually not congruent with what efficiency and productivity relate to. Efficiency and productivity measurement allows comparison of the relative performance of a given set of entities (for example hospitals) that produce the same or similar goods and/or services (for example, maternity care).

Formally, productivity is defined as the ratio of outputs to inputs and can be represented by a production frontier, with input(s) on the *(x)* axis and output(s) on the *(y)* axis. The production frontier represents the maximum output that could be produced from each input level given current technology. Firms operate either on that frontier, if they are technically efficient or beneath the frontier if they are not technically efficient. If a firm is beneath the frontier, this indicates that they could be producing more outputs then they currently are. Technical efficiency is defined as the production of the maximum amount of output from a given amount of input (or alternatively the production of a given output with minimum input quantities) given current technology. Allocative efficiency is similar to technical efficiency but places a cost on inputs or outputs. Allocative efficiency is defined as the input mix that minimizes cost, given input prices or when the output mix maximizes revenue, given output prices. Together, technical and allocative efficiency comprise overall economic efficiency [5]. Efficiency and productivity measurement places equal emphasis on inputs (or costs) and outputs (what is actually produced). If costs are reduced and what is produced also simultaneously declines, productivity is not increased, and efficiency is unlikely to be reached.

In any efficiency and productivity analysis the selection of relevant input and output measures is an essential first step. However, within healthcare capturing what is produced can be difficult. Output measures should reflect the function and key activities of a given industry and allow comparison of both the quantity and quality of output [5, 6]. For the health industry, the most relevant output variable would be one that measures the health gains of individual patients who seek treatment [6, 7]. However, there is often limited data available on individual patient outcomes. Many efficiency and productivity studies therefore utilise proxy measures of health outcomes, such as number of patients treated or length of stay.

The limitations of proxy measures of health outcomes, such as number of patients or length of stay, is further pronounced when considering the performance of maternity services and the importance of woman-centred care. Woman-centred care promotes the principles of choice, control, continuity of caregiver and self-determination [8, 9] . It is increasingly being incorporated in Australia and other jurisdictions as the foundation of the provision of safe and effective maternity care [10, 11]. Woman-centred care recognizes that a ‘successful’ birthing experience is defined by more than the delivery of a healthy baby and the physical safety of the mother. The selection of output measures to assess the efficiency and productivity of maternity services should therefore also move beyond simple measures of maternal and neonate morbidity and mortality and indicators of clinical activity, and towards those variables that capture the entirety of the birthing experience.

The aim of this paper is to identify potential output measures that reflect the principles of woman-centred care and that can be included in an assessment of the efficiency and productivity of maternity services in Australia. This paper will survey available perinatal and maternal datasets in Australia to identify potential output measures; map identified output variables against the principles of woman-centred care outlined in Australia’s national maternity strategy *Woman-centred care: Strategic Directions for Australian Maternity Services*; and based on this data, create a preliminary composite outcome measure for use in assessing the efficiency and productivity of Australian maternity services. It will then demonstrate the potential application of this output measure in creating a production frontier for hospitals in Queensland, Australia.

## Methods

### Principles of maternity care in Australia

The Australian national strategy, *Woman-centred care: Strategic Directions for Australian Maternity Services,* outlines a means to support the delivery of maternity services for women from conception until 12 months after pregnancy or birth. The Strategy outlines four values – safety, respect, choice and access – which underpin twelve principles for woman-centred maternity care that apply to all health professionals providing maternity services [12]. The twelve principles for woman-centred care and their corresponding values are shown in Table 1.

**Table 1:** The Twelve Principles for Woman-centred Maternity Care in the national maternity strategy

**The Twelve Principles for Woman-centred Maternity Care** [12]	
**Safety**	
Women receive individualised information and appropriate care during the perinatal period that is based on current, high quality evidence.	
Women have access to individualised culturally safe and responsive maternity care, in their preferred language.	
Women access care from a maternity care workforce that is responsive, competent, resourced and reflects cultural diversity.	
**Respect**	
Women are treated with dignity and respect throughout maternity care.	
Maternity care is holistic, encompassing a woman’s physical, emotional, psychosocial, spiritual and cultural needs.	
Women’s safety and experience of maternity care is underpinned by respectful communication and collaboration among health professionals.	
**Choice**	
Women are provided with and can readily access information about all locally available maternity services.	
Women are supported to make informed decisions and choices about their care.	
Women’s choices and preferences are sought and respected throughout maternity care.	
**Access**	
Women have access to appropriate maternity care where they choose from conception until 12 months after birth.	
Women have access to continuity of care with the care provider(s) of their choice — including midwifery continuity of care.	
Women have access to mental health information, assessment, support and treatment from conception until 12 months after birth	

This national strategy provides a useful framework for considering potential output variables for assessment of the efficiency and productivity of maternity services in Australia. Ideally, any efficiency and productivity analysis would incorporate output variables that correlate with and indicate how well a maternity service is delivering care in accordance with these twelve principles [12].

### Data scoping

In order to identify data sources from which to create an output measure, a search was conducted for datasets available within Australia that related to maternal health care. A Google search engine (Chrome) was used to search for and identify relevant datasets. The following keywords and phrases were included in the search: *Maternal; Maternity; Perinatal; Pregnancy; Childbirth; Data; Collection; Indicators.*

### Data mapping

Identified datasets relating to maternal health care were reviewed. The contents of each dataset was categorized as either descriptive measures of baseline demographic or clinical characteristics, ‘process measures’, or potential outcome measures. Baseline demographic details record the details of women at the start of pregnancy and are not affected by maternity care; process measures are a part of the services that are delivered to women in order to achieve an outcome. An outcome is seen to be an end product of maternity care, and not a part of the care itself. The outcome measures were then mapped to the four values of safety, respect, choice and access outlined in Australia’s national maternity strategy. From this mapping exercise a preliminary composite outcome measure was constructed for use in assessing the efficiency and productivity of Australian maternity services.

### Example of outcome measure: construction of a productivity function for Queensland maternity services

Using an existing whole-of-population linked administrative dataset [13] we applied our composite outcome measure to demonstrate the change in the Production Function if all births (quantity) were used as the output or only births that met the criteria of the composite outcome measure (quantity and quality). The linked administrative dataset covers all births in the Australian state Queensland, between 1st July 2012 and 30th June 2015. It utilizes the Queensland Perinatal Data Collection to identify births, and was then linked to the Queensland Hospital Admitted Patient Data Collection, Queensland Emergency Department Collection, and Medical Benefits Schedule and Pharmaceutical benefits Scheme (PBS) claims records. Together this data set records the cost to governments (public hospital funders, Medicare, the PBS), private health insurers and individuals through out of pocket costs [13].

To create the production function, the number of births in each hospital jurisdiction across Queensland (termed ‘Hospital and Health Service’ (HHS)) in 2014 was identified; the number of births that met the requirement of out composite measure in 2014 was then identified. The total costs to governments, private health insurers and individuals for all services accessed by the women and the child from onset of pregnancy to 12 months postpartum was then summed for all women in each HHS. This is the time period covered by the national strategy *Woman-centred care: Strategic Directions for Australian Maternity Services*. The Production Function plots the total output for each HHS on the y-axis, and the value of the total inputs (the total cost) on the x-axis to create a measure of productivity of each hospital. The difference in the production function using only total number of births, and the composite output measure was compared to demonstrate the utility of the composite output measure.

## Results

### Existing maternity and perinatal data sets in Australia

#### National core maternity indicators, National Perinatal Data Collection and state perinatal data collections

The National Core Maternity Indicators (NCMIs) provide information on measures of clinical activity and outcomes in relation to maternity care across Australia. The purpose of the indicators is to establish baseline data to monitor and evaluate maternity care in Australia and enable continuous improvement in care. The NCMIs are clinical indicators of maternity care, where a clinical indicator is defined as a measure of the clinical management and outcome of care and is based on evidence that confirms the underlying causal relationship between a particular process or intervention and health outcome [14]. The NCMIs are constructed from data items from the Australian Institute of Health and Welfare (AIHW) National Perinatal Data Collection (NPDC), a national population-based collection that provides information on the pregnancy and childbirth of mothers, and the characteristics and outcomes of their babies. The NPDC captures all births in Australia in hospitals, birth centres and the community [15].

Tables 2 and 3 identify descriptive measures, process measures and outcome measures, and for the outcome measures, map the NCMIs and NPDC data items against the four values of the national strategy – safety, respect, choice and access. Note that in undertaking this mapping exercise the focus was to identify the immediate, rather than downstream, effects of the outcome measure data items in relation to the values of safety respect, choice and access, consistent with this paper’s focus on in-hospital care for labour and birth. In addition to the specified core NPDC each state-level PDC was surveyed. The common data items in these data sources are listed in Table 4.

**Table 2:** National Core Maternity Indicators mapped to the values of the Australian national maternity strategy

Indicators	Descriptive	Process	Outcome measures				
			Safety	Respect	Choice	Access	Cost Adjustment
**Antenatal Period Indicators**							
Tobacco smoking in pregnancy: a. in the first 20 weeks of pregnancy for all women giving birth b. after the first 20 weeks of pregnancy for all women who gave birth and reported smoking during pregnancy			X				X
Antenatal care in the first trimester for all women giving birth		X					
**Labour and Birth Indicators**							
Induction of labour for selected women^a^ giving birth for the first time		X					
Caesarean section for selected women giving birth for the first time		X					
Non-instrumental vaginal birth for selected women^a^ giving birth for the first time		X					
Instrumental vaginal birth for selected women^a^ giving birth for the first time		X					
Episiotomy for women having their first baby and giving birth vaginally: a. without instruments to assist the birth b. assisted with instruments			X		X		
General anaesthetic for women giving birth by caesarean section		X					
Women having their second birth vaginally whose first birth was by caesarean section		X					
**Birth Outcome Indicators**							
Apgar score of less than 7 at 5 min for births at or after term			X				
Small babies among births at or after 40 weeks gestation			X				
Third and fourth degree tears: a. for all vaginal first births b. for all vaginal births			X				

^a^Rather than the whole population, these indicators are measured only for ‘selected women’. This is women whose characteristics indicate they have a lower risk of birth complications and therefore provide a better indication of what are expected outcomes in ‘standard’ cases. Selected women are aged between 20 and 34 years; gave birth between 37 and 41 completed weeks of gestation; had a singleton baby who presented in the vertex (head down) position [16]

**Table 3:** National Perinatal Data Collection Minimum Data Set mapped to Australian national maternity strategy values

Data Item	Descriptive	Process measure	Outcome				Cost Adjustment
			Safety	Respect	Choice	Access	
Birth event—anaesthesia administered, yes/no		X					
Birth event—analgesia administered, yes/no		X					
Birth event—birth method: Vaginal—non-instrumental; Vaginal—forceps; Caesarean section; Vaginal— vacuum extraction		X					
Birth event—birth plurality: Singleton; Twins; Triplets; Quadruplets; Quintuplets; Sextuplets; Other	X						X
Birth event—birth presentation: Vertex; Breech; Face; Brow; Other	X						
Birth event—labour onset type: Spontaneous; Induced; No labour		X					
Birth event—setting of birth (actual): Hospital, excluding birth centre; Birth centre, attached to hospital; Birth centre, free standing; Home; Other		X					
Birth event—state/territory of birth	X						X
Birth event—type of anaesthesia administered: Local anaesthetic to perineum; Pudendal block; Epidural or caudal block; Spinal block; General anaesthesia; Combined spinal-epidural block; Other anaesthesia		X					
Birth event—type of analgesia administered: Nitrous oxide; Epidural or caudal block; Spinal block; Systemic opioids; Combined spinal-epidural block; Other analgesia		X					
Birth—Apgar score (at 5 min)			X				
Birth—birth order: Singleton or first of a multiple birth; Second of a multiple birth; Third of a multiple birth; Fourth of a multiple birth; Fifth of a multiple birth; Sixth of a multiple birth; Other	X						
Birth—birth status: Live birth; Stillbirth (fetal death)			X				
Birth—birth weight, total grams			X				
Episode of admitted patient care—separation date	X						X
Establishment—organisation identifier (Australian)	X						X
Female (mother)—postpartum perineal status: Intact; 1st degree laceration/vaginal graze; 2nd degree laceration; 3rd degree laceration; Episiotomy; 4th degree laceration; Other perineal laceration, rupture or tear			X				
Female (pregnant)—number of cigarettes smoked (per day after 20 weeks of pregnancy)			X				X
Female (pregnant)—tobacco smoking indicator (after twenty weeks of pregnancy), yes/no			X				X
Female (pregnant)—tobacco smoking indicator (first twenty weeks of pregnancy), yes/no	X						X
Female—caesarean section at most recent previous birth indicator, yes/no	X						
Female—number of antenatal care visits		X					
Female—parity, total pregnancies	X						
Person—area of usual residence, statistical area level 2 (SA2) code (ASGS 2016)	X						X
Person—country of birth	X						X
Person—date of birth	X						X
Person—Indigenous status: Aboriginal but not Torres Strait Islander origin; Torres Strait Islander but not Aboriginal origin; Both Aboriginal and Torres Strait Islander origin; Neither Aboriginal nor Torres Strait Islander origin	X						X
Person—person identifier	X						X
Person—sex: Male; Female; Intersex or indeterminate	X						X
Pregnancy—estimated duration (at the first visit for antenatal care), completed weeks		X					
Product of conception—gestational age, completed weeks			X

**Table 4:** Common data items collected in state Perinatal Data Collections

Data Item	Descriptive	Process Measure	Outcome				Cost Adjustment
			Safety	Respect	Choice	Access	
Pregnancy complications (ICD-10 code)			X				
Labour and delivery complications (ICD-10 code)			X				
Admission to Special Care Nursery or Neonatal Intensive Care Nursery			X				
Neonatal morbidity (ICD-10 code)			X				

#### International consortium for Health outcomes measurement pregnancy and childbirth standard set

The International Consortium for Health Outcomes Measurement (ICHOM) is a not-for-profit organization that was established to promote and facilitate the global uptake of value-based health care. Value-based health care is a theoretical framework that places patients at the centre of care. It defines value as the ratio of outcomes of care divided by the cost of achieving those outcomes, where outcomes are defined as relevant end results of care from the perspective of the patient. To facilitate the implementation of value-based care, ICHOM works with international Working Groups of clinicians, researchers and patients to define standardized outcome measure sets (Standard Sets) for evaluating value in specific condition areas [17].

ICHOM has developed a Pregnancy and Childbirth Standard Set that identifies 24 outcome measures to evaluate care during pregnancy and up to 6 months postpartum. Specific outcome measures are grouped across four domains: Patient satisfaction with care; survival; morbidity; and patient-reported health and well-being*.* The Standard Set also includes a list of case-mix factors to allow comparison of outcomes across various patient populations. The ICHOM Pregnancy and Childbirth Set is currently being tested for routine implementation in Australia [16]. Table 5 shows the Pregnancy and Childbirth Standard Set outcomes mapped to four values outlined in Australia’s national maternity strategy, and identifies those outcomes that are more appropriately included in an efficiency and productivity analysis as cost adjustments.

**Table 5:** ICHOM Pregnancy and Childbirth Standard Set mapped to Australian national maternity strategy values

Measure	Demographic	Process measures	Outcomes				Cost Adjustment
			Safety	Respect	Choice	Access	
Maternal death			X				
Still birth			X				
Neonatal death			X				
Maternal need for intensive care			X				
Maternal length of stay		X					
Late maternal complication			X				
Transfusion		X					
Spontaneous pre-term birth		X					
Iatrogenic pre-term birth		X					
Oxygen dependence			X				
Neonate length of stay		X					
Birth injury			X				
Health related quality of life							
Incontinence			X				
Pain with intercourse			X				
Success with breastfeeding					X		
Confidence with breastfeeding				X	X		
Mother-infant attachment				X			
Confidence with role as a mother				X			
Postpartum depression							
Satisfaction with the results of care				X	X		
Confidence as an active participant in healthcare decisions				X			
Confidence in healthcare providers				X			
Birth experience				X			

### Constructing an output measure based on current data

Output measures for assessing efficiency and productivity of maternity services in Australia should ideally reflect the values and principles of woman-centred care. Australia’s national maternity strategy clearly articulates Australia’s vision for the provision of woman-centred maternity care based on the values of safety, respect, choice and access. This strategy therefore provides a useful framework to consider potential output measures for assessment of the efficiency and productivity of maternity services in Australia. Ideally, any efficiency and productivity analysis would incorporate output measures that correlate with and indicate how well a maternity service is delivering care in accordance with these values. They should also be able to be applied nationally, using data that is collected and accessible in every state and territory.

With these principles in mind, we have constructed a composite output measure that can be used in assessing the efficiency and productivity of maternity services in Australia. This composite measure consists of labour and birth outcomes indicators where data is available from currently available datasets. These measures are shown in Table 6 and are mapped against the four values of Australia’s national maternity strategy: safety; choice; respect; access.

**Table 6:** Composite output measure for assessing maternity service efficiency and productivity in Australia

Composite Measure (number of births with the absence of the following factors)	Values of women-centred maternity care			
	Safety	Respect	Choice	Access
Birth status: Stillbirth Birth	X			
Neonatal death within 60 days				
Apgar score of 7 or more at 5 min for births at or after term	X			
Admission to NICU	X			
Neonatal morbidity - Hypoxic-ischemic encephalopathy	X			
Neonatal morbidity – birth trauma	X			
Neonatal morbidity – Intrauterine hypoxia	X			
Other neonatal morbidity - meconium aspiration syndrome, congenital pneumonia or respiratory distress syndrome	X			
Maternal death – within 60 days of birth				
Mother’s postpartum perineal status: 3rd or 4th degree tear	X	X		
Mother morbidity – postpartum haemorrhage	X			
Mother morbidity – intrapartum haemorrhage	X			
Mother morbidity – ruptured uterus	X			

**Fig. 1:**
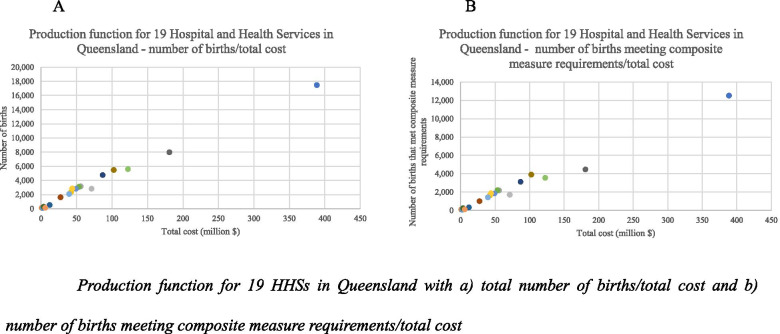
Production function for 19 HHSs in Queensland with a) total number of births/total cost and b) number of births meeting composite measure requirements/total cost

The composite measure was applied to the Queensland population of women giving birth in 2014. Table 7 shows the number of women giving birth in each HHS (quantity), and the number of births that met our composite measure requirements (quantity and quality). Figure 1, panel A shows the production function with only the number of births in each HHS, and Fig. 1, panel B shows the production function with the number produced with the application of our composite measure.

**Table.7:** Number of women giving birth in each Queensland HHS and the number of births that meet composite measure requirements

HHS	Number of births	Number of births meeting composite measure requirements
**1**	17,479	12,527
**2**	119	89
**3**	3152	2177
**4**	2361	1638
**5**	96	80
**7**	3091	2192
**8**	4758	3121
**9**	1622	1003
**10**	7976	4470
**11**	5471	3904
**12**	517	332
**13**	268	235
**14**	2816	1850
**15**	134	108
**16**	2821	1707
**17**	2863	1872
**18**	2069	1419
**19**	5602	3549
**Total**	63,215	42,273

## Discussion

It can be seen that routine data currently collected in Australia relate predominantly to process measures and outcome measures that cover issues of safety, rather than the values of respect, choice and access. Nevertheless, many of the items have some utility when considering the efficiency and productivity of maternity services. We were able to construct a composite measure that differentiated between quantity of births and quality.

The measures included in our composite outcome measure captured important information regarding the physical health of mother and baby following labour and delivery. A baby’s Apgar score assesses the clinical status of a baby immediately following childbirth. Third and fourth degree tears are classified as severe trauma to the perineum and can occur spontaneously or as a result of obstetric intervention during vaginal birth. The neonatal and maternal morbidity measures similarly relate to adverse outcomes that could be avoided with alternate care. The physical health of mother and baby is central to the provision of safe and effective maternity care and these indicators are therefore core choices for inclusion as output variables in an efficiency and productivity analysis.

The identified data sources covered a number of common medical interventions which are provided as a part of delivering care, which are not seen to be outcomes in themselves. Similarly, antenatal care in the first trimester, which is associated with better maternal health in pregnancy, fewer interventions in late pregnancy and positive child health outcomes [14], is still considered a process measure as it relates to the provision of care. Indicators such as these are more appropriately included in an efficiency and productivity analysis as measures of input, rather than output measures. Output measures should be reflective of health status and functionality related to care received or delivered.

It is notable, however, that although medical interventions in delivery are often required to ensure the safety of mother and baby, Australia is known to have a high rate of potentially unnecessary Caesarean sections, induction and episiotomy [18, 19]. This can be seen as symptomatic of the medicalisation of the birthing experience and in the context of woman-centred care there is a clear impetus to eliminate unnecessary birth interventions. These labour and birth indicators are therefore highly relevant to include in exploring drivers of cost in efficiency and productivity measurement. Maternity services with a similar casemix should exhibit a similar rate of medical intervention and thus cost.

A number of the ICHOM Pregnancy and Childbirth Standard Set measures broadly map to data items collected as part of Australia’s routine data collection, such as those related to ‘Survival’, ‘Severe maternal morbidity’ and ‘Neonatal morbidity’. However, measures related to ‘Patient-reported health status’, ‘Role transition’, ‘Satisfaction with care’ and ‘Healthcare responsiveness’ capture directly women’s experiences of pregnancy and childbirth and have no equivalencies in Australia’s national maternal data collection. Although the ICHOM Pregnancy and Childbirth Set has not been fully evaluated as a valid and reliable instrument for data collection in the health system [16] and is not utilized at a national level within Australia, a number of studies in Australia have verified its utility in measuring the mental and physical health of women during pregnancy and the postpartum period [16, 20]. Implementation of the ICHOM Pregnancy and Childbirth Set in Australia, subject to a rigorous validation process, would significantly enhance the national maternity data collection and provide a more comprehensive picture of how maternity services across the nation are delivering care in accordance with the values and principles of the national strategy. It would also allow more sophisticated and relevant analyses of the efficiency and productivity of maternity services, allowing for the inclusion of output variables that directly relate to woman’s experience of pregnancy and childbirth.

A number of states in Australia have made some attempt to collect woman-reported outcomes. Some of these surveys include questions that have a strong alignment with the national strategy values of safety, respect, choice and access. For instance the New South Wales Maternity Care survey includes questions that relate to women’s experiences of care in public hospitals during various stages of their maternity journey, from antenatal care, care during labour and birth, postnatal care in hospital and follow-up care at home [21, 22]. The Queensland Maternity Patient Experience Survey is a similar survey that also has strong alignment to the national strategy values of safety, respect, choice and access. However, both these surveys capture the experience of only a small number of women (the 2017 New South Wales Survey represents only 8% of the approximately 62,000 women who gave birth in one of 71 New South Wales public hospitals in 2017) [23, 24]. Other jurisdictions in Australia also have in place surveys to measure patient experience. Western Australia [25] and South Australia [26] employ randomized surveys to collect and measure data regarding patient experience, but none of these surveys relate specifically to consumers of maternity care. Victoria also employs randomized surveys to measure patient experience, but also includes specialized questionnaires for maternity clients. However, none of these surveys are as comprehensive or as widely reported as the New South Wales or Queensland surveys. These state-based patient experience surveys clearly show the limited nature of data collection in Australia regarding woman-reported outcomes. They demonstrate that there is a clear need for a national survey that includes newly developed indicators that map specifically to the values of safety, respect, choice and access.

## Conclusion

The composite measure developed in this paper makes it very clear that there are significant gaps in Australia’s maternity data collections with regard to measuring how well a maternity service is performing against the values of respect, choice and access. Adoption in Australia of the collection of woman-reported maternity outcomes would substantially strengthen Australia’s national maternity data collections and provide a more holistic view of pregnancy and childbirth in Australia beyond traditional measure of maternal and neonate morbidity and mortality. There is an urgent need for the development and implementation of national indicators that include woman-reported outcomes and which align with the values of safety, respect, choice and access and the twelve principles of woman-centred care outlined in the national maternity strategy. This would go some way to providing a more comprehensive and nuanced assessment of how well Australian maternity services are performing in the delivery of women-centred care. It would also provide a substantial foundation upon which to develop a sophisticated analysis of the efficiency and productivity of maternity services in Australia and their performance in providing woman-centred care as outlined in the national maternity services strategy.

## Data Availability

Not applicable. Data sharing is not permitted under our ethics approval.
